# Cardiomyocyte Inflammasome Signaling in Cardiomyopathies and Atrial Fibrillation: Mechanisms and Potential Therapeutic Implications

**DOI:** 10.3389/fphys.2018.01115

**Published:** 2018-08-13

**Authors:** Gong Chen, Mihail G. Chelu, Dobromir Dobrev, Na Li

**Affiliations:** ^1^Section of Cardiovascular Research, Department of Medicine, Baylor College of Medicine, Houston, TX, United States; ^2^Comprehensive Arrhythmia Research and Management Center, School of Medicine, University of Utah, Salt Lake City, UT, United States; ^3^Cardiovascular Medicine Division, Section of Cardiac Electrophysiology, School of Medicine, University of Utah, Salt Lake City, UT, United States; ^4^Institute of Pharmacology, West German Heart and Vascular Center, University Duisburg-Essen, Essen, Germany; ^5^Cardiovascular Research Institute, Baylor College of Medicine, Houston, TX, United States; ^6^Department of Molecular Physiology and Biophysics, Baylor College of Medicine, Houston, TX, United States

**Keywords:** NLRP3 inflammasome, innate immune system, cardiomyocytes, cardiac fibroblasts, cardiomyopathy, atrial fibrillation

## Abstract

Inflammasomes are high molecular weight protein complexes in the cytosol of immune and other cells that play a critical role in the innate immune system in response to cellular stress. NLRP3 inflammasome, the best-understood inflammasome, is known to mediate the maturation (activation) of caspase-1 from pro-caspase-1, causing the maturation and release of cytokines (e.g., interleukin-1β) and potentially leading to a form of inflammatory programmed cell death called pyroptosis. Previous work has shown that the NLRP3 components are expressed in cardiomyocytes and cardiac fibroblasts and recent studies have identified the NLRP3 inflammasome as a key nodal point in the pathogenesis of cardiomyopathies and atrial fibrillation, which may create an opportunity for the development of new therapeutic agents. Here we review the recent evidence for a role of NLRP3 inflammasome in the cardiomyocytes and discuss its potential role in the evolution of cardiac remodeling and arrhythmias and new opportunities created by these very recent developments.

## The Innate Immune System, Inflammation and Inflammasome Signaling

Inflammation is a vital biological process involving an acute response to infection and tissue damage aiming to prevent harmful influence to the host (Medzhitov, 2008; Buckley et al., 2013). The mammalian innate immune system plays an important role in recognizing foreign pathogen- or damage-associated molecular patterns (PAMPs and DAMPs, respectively) and defending the host against infection or injury caused by other pathological organisms (Matzinger, 1994).

**FIGURE 1:**
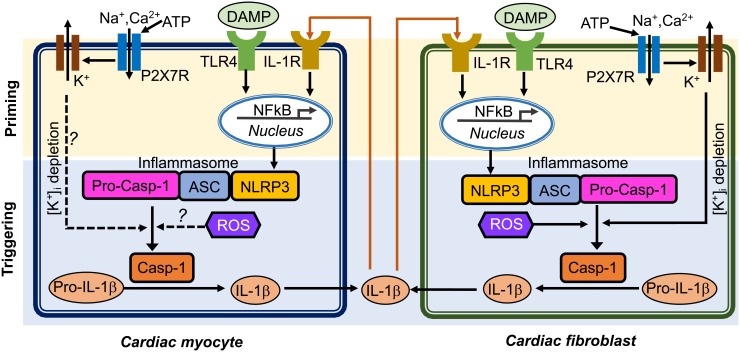
Putative mechanisms of NLRP3 inflammasome activation in cardiomyocytes and cardiac fibroblasts. Question marks indicated the mechanisms that need to be investigated. ASC, apoptosis-associated speck-like protein containing a CARD; ATP, adenosine triphosphate; Casp-1, caspase-1; DAMP, damage-associate molecular pattern; IL-1β, interleukin-1β; IL-1R, interleukin-1 receptor; NFκB, nuclear factor kappa-light-chain-enhancer of activated B cells; NLRP3, NACHT, LRR and PYD domain containing protein 3; ROS, reactive oxidative species; pro-Casp-1, precursor Caspase-1; P2X7R, P2X7 receptor; TLR4, toll-like receptor 4.

Inflammasome acts as an intracellular innate immune sensor (Martinon et al., 2002). The inflammasome is a multi-protein signaling platform that generally involves 3 proteins: (1) a NOD-like receptor (NLR), (2) an adaptor protein like apoptosis-associated speck-like protein containing a CARD (ASC), and (3) a cysteine protease such as caspase-1 or caspase-5 (Kanneganti, 2015). NLRs are a class of pattern recognition receptors (PRRs) that act as a sensor for the inflammasome. At least 22 different NLR proteins have been identified in humans and 34 in mouse (Ting et al., 2008). Most NLRs consist of a tripartite structure that includes: (1) a N-terminal caspase-recruitment domain (CARD) or pyrin domain that mediates downstream protein-protein assembly, (2) a centrally located nucleotide-binding-and-oligomerization domain that facilitates self-oligomerization, and (3) a C-terminal leucine-rich repeats (LRRs) that are thought to be involved in stimuli sensing (Martinon et al., 2002). To date, the best investigated and validated inflammasome type is the “NACHT, LRR and PYD domain containing protein 3” (NLRP3) inflammasome (He et al., 2016). Upon recognizing a series of inflammation-inducing stimuli (e.g., PAMPs and DAMPs), NLRP3 inflammasomes in the innate immune cells activate caspase-1 (Casp-1) which promotes the production of proinflammatory cytokines (IL-1β and pro-IL-18) and may lead to cell death known as pyroptosis (Schroder and Tschopp, 2010; Davis et al., 2011). The activation of the NLRP3 inflammasome requires two primary signals (**Figure 1**): (1) a priming step in which the toll-like receptor (TLR)-nuclear factor-κB (NFκB) signaling pathway promotes the transcription of NLRP3 and precursor ILs (pro-IL1β or pro-IL-18); and (2) a triggering step in which a series of stimuli (K^+^ efflux, increase in cytosolic Ca^2+^, generation of reactive oxygen species [ROS], mitochondrial dysfunction, and lysosomal rupture) (He et al., 2016) can facilitate the assembly of the NLRP3 inflammasome components by recruiting precursor-caspase-1 (pro-Casp-1) into the complex via the adaptor protein ASC. This promotes the autocleavage of pro-Casp-1 to Casp-1 containing the active p20/p10 heterodimer proteins (Wilson et al., 1994). Alternatively, NLRP3 inflammasome may also activate caspase-11 or human orthologues caspase-4/caspase-5 (casp-4/5), which is also known as “non-canonical” NLRP3 inflammasome pathway (Kanneganti, 2015). Activated Casp-1 holoenzyme further cleaves pro-IL-1β and pro-IL-18 to form their respective mature forms (Li et al., 1995; Gu et al., 1997). Mature IL-1β is a potent proinflammatory mediator in many immune reactions, including the recruitment of innate immune cells to the site of infection and modulation of adaptive immune cells, whereas mature IL-18 is important for the production of interferon-γ and potentiation of cytotoxic activity of natural killer and T cells (Dinarello, 2009).

In addition, active Casp-1 may promote pyroptosis, which is characterized by increased membrane permeability with extracellular release of pro-inflammatory signaling molecules and cell swelling and eventually cell membrane rupture (Fink et al., 2008; Toldo and Abbate, 2018). Oligomerization of N-terminal fragments resulting from the cleavage of gasdermin D by Casp-1 lead to formation of membrane pores, which are permeable for mature IL-1β, IL-18, and active Casp-1 (Liu et al., 2016). Furthermore, cleavage of several proteins involved in the Krebs cycle by Casp-1 leads to a significant decrease in cell energy production which results in cell swelling and rupture (Shao et al., 2007).

## NLRP3 Inflammasome Signaling in the Heart

Cardiomyocytes (CMs) make up approximately 75% of normal adult myocardial tissue (Camelliti et al., 2005). Although non-cardiomyocytes occupy a relatively small fraction of myocardial volume, they are essential for normal cardiac function by providing extracellular matrix (ECM), intercellular communication, and vascular supply needed for efficient function and survival of CMs (Travers et al., 2016). The NLRP3 inflammasome components have been identified in both CMs and cardiac fibroblasts (CFs), which are the two most abundant cell populations in the mammalian heart (Baudino et al., 2006). An augmented function of NLRP3 inflammasome has been proposed to play a role in multiple human diseases, such as auto-inflammatory disease (Hoffman and Wanderer, 2010), diabetes (Vandanmagsar et al., 2011), atherosclerosis (Duewell et al., 2010; Baldrighi et al., 2017), and ischemic cardiomyopathy (Kawaguchi et al., 2011; Mezzaroma et al., 2011; Sandanger et al., 2013; Liu et al., 2014; Toldo et al., 2016). The innate immune system functions as the primary cardiac defense against pathogens and tissue damage (Askevold et al., 2014). Myocardial infarction is the most common cause of cardiac injury (Jennings et al., 1990), resulting from coronary atherosclerosis-mediated plaque rupture and involving acute loss of CMs. Necrotic cardiac cells can trigger inflammatory cascades to get rid of dead cell debris in the infarcted area (Pfeffer and Braunwald, 1990; Opie et al., 2006). Conversely, cell death can also release intracellular components, which further stimulate innate immune mechanisms to facilitate the inflammatory responses. Endogenous ligands released after injury can be recognized as danger signals by cell surface receptors, thereby activating cellular inflammatory signaling (Beg, 2002). TLR-mediated pathways can trigger post-infarction inflammatory responses by activating toll-like receptor (TLR)-nuclear factor-kB (NFκB) and related signaling (Lawrence, 2009). Chemokines recruit inflammatory leukocytes to the infarcted area, and cytokines promote leukocyte-endothelial cell adhesions. Moreover, transforming growth factor-β (TGF-β) and interleukin-10 (IL-10) can promote cardiac repair by suppressing inflammation, enhancing fibroblast-to-myofibroblast transition, and promoting ECM deposition (Kaur et al., 2009; Frangogiannis, 2014). Compared to the well-established canonical function of the NLRP3 inflammasome in the innate immune cells, the putative role of the NLRP3 inflammasome in non-immune cells including cardiac cells is poorly defined. NLRP3 and other important components of the inflammasome may not be constitutively expressed in healthy mouse and human heart but expression is induced in leukocytes, endothelial cells, CFs in the granulation tissue and CMs in the infarct border zones in a mouse model of acute MI (Yin et al., 2009; Mezzaroma et al., 2011). NLRP3, IL-1β, and IL-18 mRNA levels were shown to be increased in both left ventricle CMs and CFs in a post-MI mouse model (Sandanger et al., 2013). ASC is constitutively expressed in mouse CMs and CFs (Kawaguchi et al., 2011). The entire signaling cascade appears to be operative in CFs: NLRP3 inflammasome activation by DAMP molecule ATP, TLR ligand specific activation in a NFκB dependent manner, assembly of the NLRP3/ASC inflammasome, and activation of Casp-1 (Kawaguchi et al., 2011; Sandanger et al., 2013). ASC was highly expressed in the inflammatory infiltrate cells and weakly expressed in CMs and the interstitial cells obtained from patients who had died after an acute myocardial infarction (Kawaguchi et al., 2011). **Figure 1** illustrates the putative mechanisms potentially underlying the activation of the NLRP3 inflammasome and the postulated interaction patterns between CMs and CFs through complex autocrine and paracrine mechanisms (**Figure 1**). In this review, we will focus on the very recently established role of the NLRP3 inflammasome in cardiac cells and its potential involvement in cardiac diseases, such as cardiomyopathies and atrial fibrillation (AF).

## Role of NLRP3 Inflammasome in Cardiomyopathies

Cardiomyopathies are cardiac diseases that severely impact patient morbidity and mortality (Wexler et al., 2009). They can be induced by myocardial injury that is often accompanied by transient or persistent local inflammatory responses. This type of inflammation is deemed as sterile inflammation due to the lack of a microbial pathogen. A number of studies have illustrated a central role of NLRP3 inflammasome in murine models of ischemic and non-ischemic cardiomyopathies (Mezzaroma et al., 2011; Bracey et al., 2013; Liu et al., 2014; Toldo et al., 2016; Valle Raleigh et al., 2017). NLRP3 inflammasome can be activated by several signals generated during the initial ischemia-induced myocardial injury: dsDNA, RNA, and ATP released from dying cells. DAMPs including dsDNA and RNA can activate the TLR-NFkB signaling pathway and promote the “priming” of NLRP3 and pro-IL1β. On the other hand, ATP can activate P2X purinoceptor 7 (P2X7R) in CMs thereby enhancing the K^+^ efflux and subsequently facilitating the assembly of NLRP3 inflammasome complex, which promotes the autocatalytic activation of Casp-1. The mature Casp-1 further perpetuates myocardial remodeling via two established mechanisms. On one hand, mature Casp-1 can increase the production of IL-1β and IL-18 by cleaving their precursor proteins into the mature forms. An increased release of IL-1β and IL-18 will spread and amplify the local inflammation and promote fibrosis, a major factor contributing to the structural remodeling of myocardium (Nguyen et al., 2017). On the other hand, mature Casp-1 cleaves gasdermin-D (GSDMD), another crucial component of the NLRP3 inflammasome, resulting in the formation of the N-terminal proteolytic fragment of GSDMD (GSDMD-Nt), which can promote inflammatory cell death known as “pyroptosis” (He et al., 2015; Shi et al., 2015), further deteriorating the function of the remaining myocardium. Moreover, GSDMD-Nt is not only a potential executor of pyroptosis, but is also a requirement for the release of IL-1β, because genetic deletion of GSDMD precluding the GSDMD membrane pore formation eliminates the ability of the cells to release IL-1β (He et al., 2015; Sborgi et al., 2016). Although it is unclear which degree of NLRP3 inflammasome activation is associated with pyroptosis induction, Mezzaroma et al. (2011) has demonstrated that the Casp-1-mediated cell death is restricted to the granulation tissue and CMs located to the infarct border zone following acute myocardium infarction.

The NLRP3 inflammasome might also be implicated in the pathogenesis of non-ischemic cardiomyopathy. Bracey et al. have shown that the development of cardiac hypertrophy, apoptosis and ventricular dilatation in the cardiac-specific calcineurin transgenic mice (CN-Tg) (Bracey et al., 2013) is associated with increased protein levels of NLRP3 in cardiac tissue and IL-1β in serum, which suggest that both the “priming” and “triggering” steps are activated during the development of heart failure with reduced ejection fraction (HFrEF). Most importantly, administration of IL-1 receptor antagonist (IL-1-ra) prevented the progressive reduction of cardiac contractility, reduced infiltration of inflammatory cells in the myocardium, and decreased apoptosis in CN-Tg mice.

The pathogenesis of diabetic cardiomyopathy has also been linked to the activation of NLRP3 inflammasome (Shaw et al., 2010). Diabetic cardiomyopathy often manifests as heart failure with preserved ejection fraction (HFpEF), and is a consequence of increased ventricular wall stiffness leading to left ventricular diastolic dysfunction. It is well established that hyperglycemia increases the production of ROS, which is a known trigger for the assembly of the NLRP3 inflammasome complex. Glucose itself has also been reported to be an effective activator of the NLRP3 inflammasome (Shi et al., 2015; Zu et al., 2015). In a type-2 diabetic rat model induced by high fat diet and low dose streptozotocin, the expression of NLRP3, ASC, Casp-1, and IL-1β was increased in the heart. Genetic inhibition of Nlrp3 by a small interfering RNA *in vivo* improved left ventricular diastolic function in these diabetic rats (Luo et al., 2014), which was attributed to a reduction in cell death, an improvement of myofilament and mitochondria structures, and a reduction in cardiac fibrosis. Thus there is accumulating evidence pointing to a critical role of NLRP3 inflammasome activation in ischemic and non-ischemic cardiomyopathy.

## Role of NLRP3 Inflammasome in Af

AF is the most frequent clinical arrhythmia which is associated with an increased risk of stroke and worsening heart failure (Andrade et al., 2014; Freeman et al., 2017). The development of AF often involves ectopic triggers acting on an arrhythmogenic substrate to initiate AF-maintaining reentry (Heijman et al., 2014). The current therapeutic approaches are moderately effective perhaps because of critical gaps in current knowledge about arrhythmia mechanisms and important translational challenges of available therapeutic concepts (Heijman et al., 2016, 2018).

An enhanced inflammatory response is frequently associated with AF development (Aviles et al., 2003; Harada et al., 2015) and increased levels of circulating IL-1β and IL-18 positively correlated with progression from paroxysmal AF (pAF) to long-lasting persistent AF (perAF), along with left atrial dilatation (an independent risk factor of AF) in AF patients (Luan et al., 2010; Gungor et al., 2013). We recently showed that the activity of the NLRP3 inflammasome is increased in CMs from patients with pAF and perAF (Yao et al., 2018). In atrial CMs from pAF patients, protein levels of active Casp-1-p20 were significantly increased, despite the unchanged protein levels of NLRP3 and pro-Casp-1, likely reflecting the fact that the increase in NLRP3 inflammasome activity in pAF might be due to an increased “triggering” (enhanced assembly), rather than “priming” (increased gene transcription) processes. In contrast, atrial CMs from perAF patients showed not only higher protein levels of Casp-1-p20, but also an upregulation of NLRP3, ASC, and pro-Casp-1 proteins, indicating that both “priming” and “triggering” processes contribute to the activation of the NLRP3 inflammasome in CMs of these patients. To the best of our knowledge this study is the first to show that the NLRP3 inflammasome is expressed and upregulated in non-immune cardiac cells (CMs) from pAF and perAF patients and that its activity in human CMs correlates with the progression of AF to more persistent forms.

**FIGURE 2:**
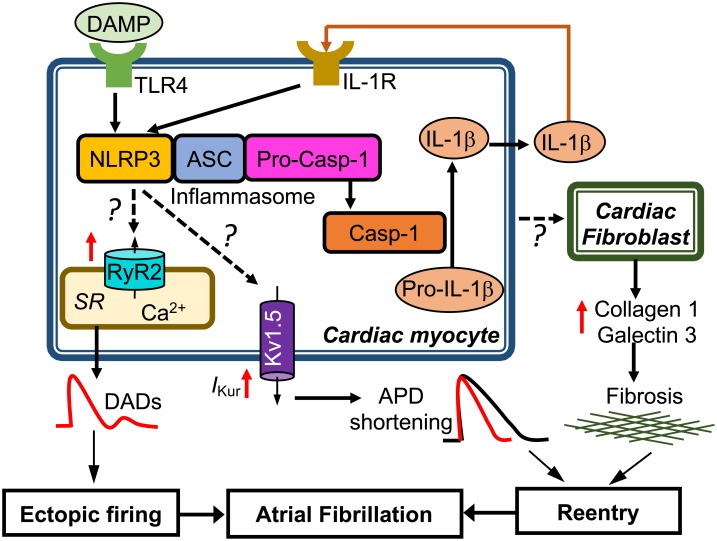
Putative molecular mechanisms underlying the development of atrial fibrillation due to constitutive activation of NLRP3 inflammasome in mouse cardiac myocytes only. Question marks indicated the mechanisms that need to be investigated. APD, action potential duration; DADs, delayed afterdepolarizations; *I*_Kur_, ultra-rapid delayed rectifier potassium current; SR, sarcoplasmic reticulum.

To determine whether CM-restricted activation of the NLRP3 inflammasome plays a causative role in AF pathogenesis, a CM-specific knockin mouse model expressing a gain-of-function mutation of NLRP3 (NLRP3^A350V^) mimicking the constitutive NLRP3 activation seen in CMs from AF patients, was established by crossing a previously established conditional allele (Brydges et al., 2009) to the CM-specific Cre transgenic mouse (*Myh6^Cre^:Nlrp3^A350V/+^*, CM-KI). In this CM-KI mouse model, total protein levels of NLRP3, ASC and pro-Casp-1 remained unchanged, whereas Casp-1-p20 protein levels were increased, recapitulating the changes we observed in pAF patients. Electrophysiological studies have demonstrated that the constitutive activation of NLRP3 inflammasome in CMs only increased the AF susceptibility by producing both ectopic (triggered) activity and reentry-promoting electrical remodeling in CM-KI mice (Yao et al., 2018). Moreover, the enhanced AF susceptibility was associated with abnormal diastolic sarcoplasmic reticulum (SR) Ca^2+^ releases due to increased protein levels of ryanodine receptor type-2 (RyR2), which might represent the molecular correlates of ectopic activity as reflected by the higher incidence of premature atrial contractions. In addition, the atrial effective refractory period (AERP) was abbreviated most likely because of an enhanced function of the ultra-rapid delayed-rectifier K^+^-current (Kv1.5) in CMs. Genetic inhibition of Nlrp3 in CM-KI mice using the adeno-associated virus type 9 (AAV9)-mediated gene transfer of a short-hairpin RNA (shRNA), reduced the incidence of inducible AF episodes. Thus, this study clearly validated the causal relationship between the CM-specific NLRP3 inflammasome activation and the susceptibility to AF (Yao et al., 2018). **Figure 2** summarizes the putative molecular mechanisms associated with AF development due to the activation of the NLRP3 inflammasome in CMs only. Since NLRP3 inflammasomes exist also in CFs and CFs play an important role in atrial fibrosis, a well-recognized substrate for AF maintenance, future studies should address the potential role of the CF NLRP3 inflammasome for AF pathophysiology.

## Targeting of the NLRP3 Inflammasome as a Therapeutic Option

Based on available experimental evidence, targeting the NLRP3 inflammasome-signaling pathway may represent a unique therapeutic opportunity for patients with cardiomyopathy and/or AF. Most of the agents described below have been tested only pre-clinically in animal models and their potential translation into clinical practice will require prospective clinical trials in the suitable patient populations. The multiple players involved in the NLRP3 inflammasome signaling cascade offer a variety of viable options for therapeutic exploitation. Targeting the upstream regulators of NLRP3 inflammasome function, selective inhibition of different members of NLRP3 inflammasome complex, interruption of the complex preventing maturation of Casp-1, and selective blockade of the downstream effectors (mature caspase-1 and IL-1β) of NLRP3-inflammasome activation, may all be viable therapeutic interventions. An inhibition of the upstream regulators could be achieved by inhibition of K^+^ efflux with the antidiabetic drug glyburide (Haque et al., 2016) or the application of a ROS-scavenger (Liu et al., 2014). The inhibition of individual NLRP3 inflammasome members could be accomplished by genetic silencing of Nlrp3, ASC, or pro-Casp-1 using specific shRNAs (Dai et al., 2011; Yao et al., 2018) or CRISP/Cas9-mediated non-homologous end joining (NHEJ) (Zhang et al., 2014) via the AAV-mediated gene transfer system. Disruption of inter–domain interaction between NLRP3 and ASC can be achieved using the MCC950 compound (Coll et al., 2015). The blockade of IL-1β functions is currently the most advanced strategy with two agents being clinically available: the IL-1 receptor antagonist anakinra (Mitroulis et al., 2010) and the neutralizing IL-1β antibody canakinumab (Jesus and Goldbach-Mansky, 2014; Ridker et al., 2017). Moreover, several Casp-1 inhibitors (e.g., Ac-WEHD-Cho and Ac-YVAD-cho) are currently under development for patients with auto-inflammatory diseases (Howley and Fearnhead, 2008; MacKenzie et al., 2010). Interestingly, the recently completed large-scale clinical trial “The Canakinumab Anti-inflammatory Thrombosis Outcomes Study” (CANTOS) showed that selective targeting of the IL-1β pathway can significantly reduce the rate of recurrent cardiovascular events including myocardial infarction, and that this effect was independent of lipid levels lowering (Ridker et al., 2017). This study provides a promise that targeting of the NLRP3-inflammasome could potentially be a viable therapeutic option for cardiomyopathies, AF, and perhaps other cardiovascular diseases including heart failure. Prospective randomized clinical trials including suitable clinical patient populations are needed to prove and validate the therapeutic potential of NLRP3 inflammasome inhibition for the management of cardiovascular diseases.

## Author Contributions

NL designed the study. GC organized the database. GC and MC wrote sections of the manuscript. DD and NL revised the manuscript critically for important intellectual content. All authors contributed to the manuscript revision, read, and approved the submitted version.

## Conflict of Interest Statement

The authors declare that the research was conducted in the absence of any commercial or financial relationships that could be construed as a potential conflict of interest.
